# Hypothalamic-Pituitary-Gonadal Endocrine System in the Hagfish

**DOI:** 10.3389/fendo.2013.00200

**Published:** 2013-12-30

**Authors:** Masumi Nozaki

**Affiliations:** ^1^Sado Marine Biological Station, Faculty of Science, Niigata University, Sado, Japan

**Keywords:** hagfish, agnathan, cyclostomes, HPG axis, pituitary gland, gonadotropin, GnRH, estradiol

## Abstract

The hypothalamic-pituitary system is considered to be a seminal event that emerged prior to or during the differentiation of the ancestral agnathans (jawless vertebrates). Hagfishes as one of the only two extant members of the class of agnathans are considered the most primitive vertebrates known, living or extinct. Accordingly, studies on their reproduction are important for understanding the evolution and phylogenetic aspects of the vertebrate reproductive endocrine system. In gnathostomes (jawed vertebrates), the hormones of the hypothalamus and pituitary have been extensively studied and shown to have well-defined roles in the control of reproduction. In hagfish, it was thought that they did not have the same neuroendocrine control of reproduction as gnathostomes, since it was not clear whether the hagfish pituitary gland contained tropic hormones of any kind. This review highlights the recent findings of the hypothalamic-pituitary-gonadal endocrine system in the hagfish. In contrast to gnathostomes that have two gonadotropins (GTH: luteinizing hormone and follicle-stimulating hormone), only one pituitary GTH has been identified in the hagfish. Immunohistochemical and functional studies confirmed that this hagfish GTH was significantly correlated with the developmental stages of the gonads and showed the presence of a steroid (estradiol) feedback system at the hypothalamic-pituitary levels. Moreover, while the identity of hypothalamic gonadotropin-releasing hormone (GnRH) has not been determined, immunoreactive (ir) GnRH has been shown in the hagfish brain including seasonal changes of ir-GnRH corresponding to gonadal reproductive stages. In addition, a hagfish PQRFamide peptide was identified and shown to stimulate the expression of hagfish GTHβ mRNA in the hagfish pituitary. These findings provide evidence that there are neuroendocrine-pituitary hormones that share common structure and functional features compared to later evolved vertebrates.

## Introduction

**Figure 1:**
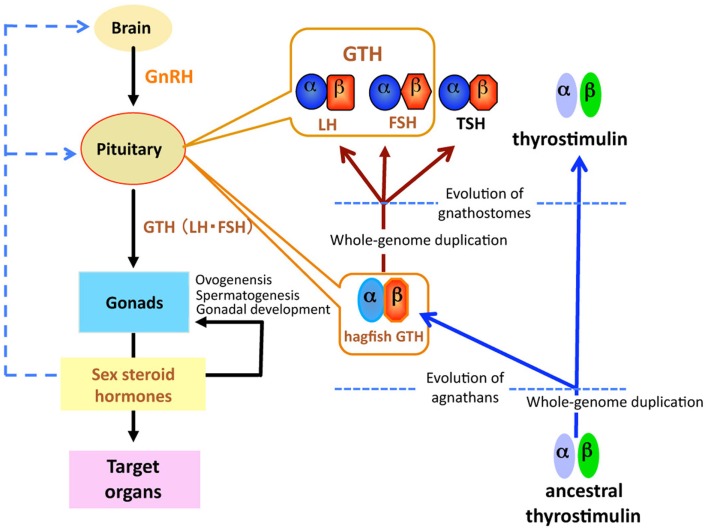
**Schematic diagram of the evolution of glycoprotein hormones in the hypothalamic-pituitary-gonadal axis**. Ancestral thyrostimulin (α and β) existed before divergence of vertebrates. An ancestral thyrostimulin (α and β) diverged into GTH (α and β) and thyrostimulin (α and β) during the early phase of agnathan divergence. The GTH (α and β) formed a heterodimer in the pituitary and acted as the first adenohypophysial gonadotropic hormone during the evolution of agnathan species. This GTH dimer further diverged into three functional units of adenohypophysis, LH and FSH as two gonadotropins, and TSH as a thyrotropin, in the lineage to gnathostomes.

Reproduction in gnathostomes (jawed vertebrates) is controlled by a hierarchically organized endocrine system called the hypothalamic-pituitary-gonadal (HPG) axis (1). In spite of the diverged patterns of reproductive strategies and behaviors within this taxon, this endocrine network is remarkably conserved throughout gnathostomes. In response to hypothalamic gonadotropin-releasing hormone (GnRH), gonadotropins (GTHs) are secreted from the pituitary and stimulate the gonads, where they induce the synthesis and release of sex steroid hormones, which in turn elicit growth and maturation of the gonads (Figure 1).

The pituitary gland is present in all vertebrates from agnathans (jawless fishes) to mammals and consists of the same two principal elements, the neurohypophysis and adenohypophysis. The neurohypophysis develops from the floor of the diencephalon as an infundibular extension, whereas the adenohypophysis develops from the epithelium that comes in contact with this infundibulum. The enigma of the pituitary gland is that evolution of a composite organ with such a complex double developmental origin must have been associated with some functionally adaptive value. Yet demonstration of this adaptive value in the agnathans themselves remains elusive. Most surprising facts are that not only the pituitary gland but also all major adenohypophysial hormones such as GTHs, growth hormone (GH), prolactin, and adrenocorticotropin (ACTH) and their receptors are also considered to be vertebrate novelties (2). Thus, the hypothalamic-pituitary system, which is specific to vertebrates, is considered to be a seminal event that emerged prior to or during the differentiation of the ancestral agnathans. Such an evolutionary innovation is one of the key elements leading to physiological divergence, including reproduction, growth, metabolism, stress, and osmoregulation in subsequent evolution of gnathostomes.

Lampreys and hagfish are the only two extant representatives of agnathans. Paleontological analysis of extinct agnathans had suggested that lampreys were more closely related to gnathostomes than either group is to the hagfishes (3, 4). However, both recent molecular phylogenetic analyses (5–7) and developmental study on the craniofacial pattern of the hagfish (8) strongly support the monophyly of the cyclostomes (lampreys and hagfishes as closest relatives). Therefore, studies on reproduction of the cyclostomes are important for understanding the evolution of the HPG axis related to vertebrate reproduction. Findings from many molecular, biochemical, physiological, and morphological studies indicate that the HPG axis is present in the lamprey [for review, see Ref. (1)]. In contrast, endocrine regulation of reproduction in the hagfish is poorly understood [for reviews, see Ref. (9, 10)]. For example, until the recent identification of functional GTH in the brown hagfish, *Paramyxine atami* (11), it was not established whether the hagfish pituitary gland contains tropic hormones of any kind. Herein, this report summarizes the recent findings of the HPG endocrine system involved in reproduction in hagfish.

## Hagfish Pituitary Gland

**Figure 2:**
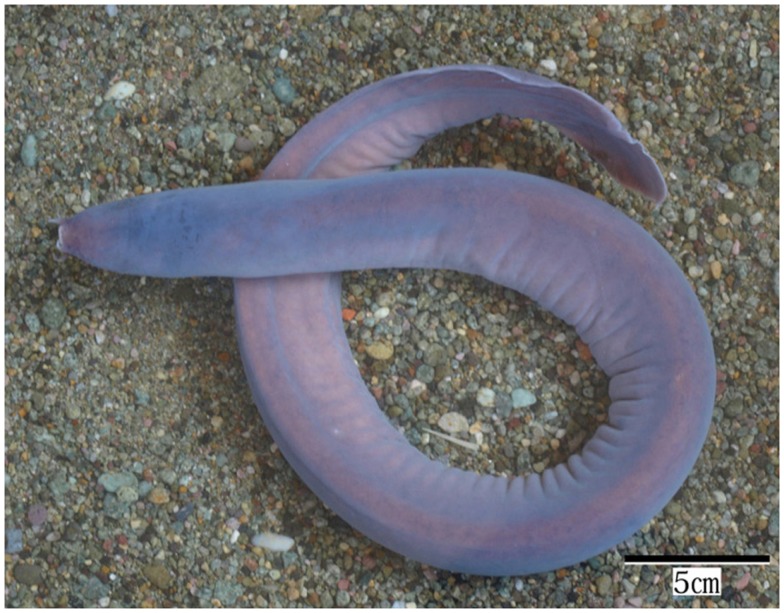
**Brown hagfish, *Paramyxine atami***.

**Figure 3:**
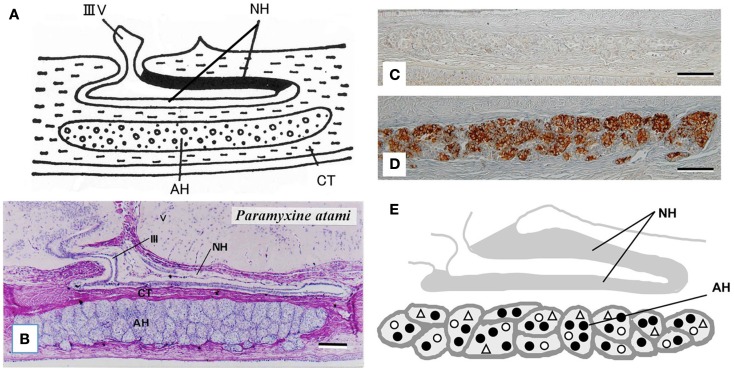
**(A)** Diagrammatically sagittal section of the hagfish pituitary gland. Dark area of the neurohypophysis (NH) shows posterior part of the dorsal wall, where ir-GnRH nerve fibers and AVT nerve fibers are densely accumulated [for AVT, see Ref. (82)]. **(B)** Nearly mid-sagittal section of the pituitary gland of the brown hagfish, stained with hematoxylin and eosin. **(C,D)** GTHβ-like immunoreaction in the adenohypophysis of the juvenile **(C)** and sexually mature **(D)** brown hagfish stained with anti-hagfish GTHβ. Note that GTH-positive cells are almost absent in **(C)**, whereas they are abundant in **(D)**. **(E)**, Diagrammatically sagittal section of the hagfish pituitary gland showing the topographic distribution of adenohypophysial cells. Closed circle, GTH cell; open circle, ACTH cell; open triangle, undifferentiated cell and possible GH cell. AH, adenohypophysis; CT, connective tissue; IIIV, third ventricle. Scale bars: 100 μm.

**Figure 4:**
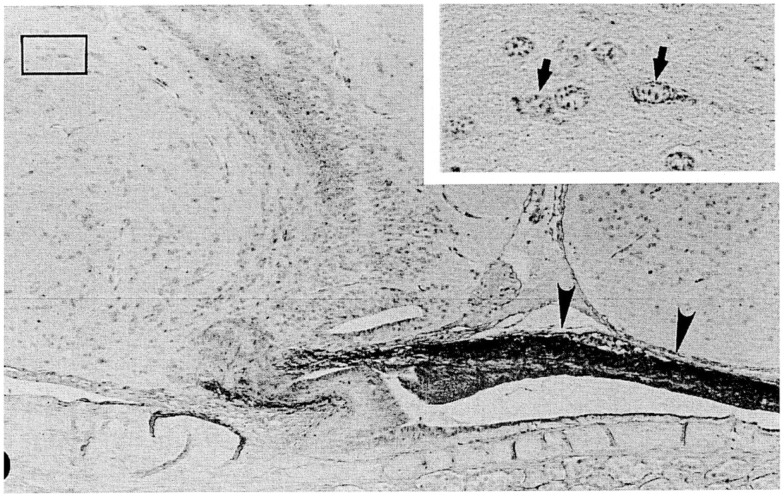
**A nearly mid-sagittal section through the neurohypophysis of the Atlantic hagfish, *Myxine glutinosa*, showing the accumulation of ir-GnRH in the dorsal wall of the neurohypophysis (arrowheads)**. This section was stained with anti-salmon GnRH. Inset, an enlargement of the rectangular area showing GnRH-positive neuronal cells. Arrows show GnRH-positive cell bodies. Scale bars: 100 μm; inset, 20 μm. From Oshima et al. (21).

The hagfish is considered the most primitive vertebrate known, living or extinct (3) (Figure 2). In addition to their primitive external body features, hagfish possess the most primitive hypothalamic-pituitary system among the vertebrates (12). The neurohypophysis is a flattened sac-like structure, whereas the adenohypophysis consists of a mass of clusters of cells embedded in connective tissue below the neurohypophysis (12, 13) (Figures 3A,B). The adenohypophysis and the neurohypophysis are completely separated by a layer of connective tissue, and there is no or little anatomical relationship between them (14, 15) (Figure 3B). In addition, there is no clear cytological differentiation between the pars distalis and the pars intermedia (12, 13) (Figure 3B). The question arises whether the simplicity of the hagfish pituitary gland is a primitive or a degenerate feature. For example, some authors have claimed that the pars intermedia seems to have been lost via a secondary degenerative process (13, 16). Moreover, until recent identification of a functional GTH in the hagfish pituitary (11), it had not been established whether the hagfish pituitary gland contained adenohypophysial hormones of any kind (9). Because of the simplicity and primitiveness of the pituitary morphology and equivocal data on the adenohypophysial hormones in the hagfish, many researchers had questioned whether there were any functional adenohypophysial hormones (9, 17). On the other hand, arginine vasotocin (AVT), as a single neurohypophysial hormone, was identified in the hagfish (18). In addition, the presence of GnRH has been suggested in the hagfish hypothalamus by both radioimmunoassay (RIA) and immunohistochemistry (19–22) (Figure 4). Thus, the hagfish appears to have neurohypophysial and hypothalamic hormones similar to those of other vertebrates.

At present, the adenohypophysis of the hagfish is the least understood of all the vertebrates. However, our immunohistochemical studies provided the first clear-cut evidence for the presence of GTH and ACTH in the hagfish (23–25). Although not conclusive, our data also suggested the presence of GH in the hagfish (23). In addition, these three adenohypophysial hormones were suggested to be the ancestral adenohypophysial hormones that have maintained their original functions throughout vertebrate evolution. On the other hand, the later derived hormones, such as prolactin and thyroid-stimulating hormone (TSH), may have contributed to the expansion of vertebrates into new environments, as suggested by Kawauchi et al. (26) and Kawauchi and Sower (27). Moreover, our study further revealed that GTH cells, ACTH cells, and unidentified cells which were assumed to include both undifferentiated cells and GH cells, were packed together in the same cell cluster of the hagfish adenohypophysis, and thus each cluster appeared to serve as a separate functional unit (10, 24) (Figures 3C–E). If the absence of the pars intermedia is the most ancestral vertebrate pituitary gland, melanophore-stimulating hormone (MSH) activity seems to be gained secondarily together with the differentiation of the pars intermedia. Further studies are needed to clarify this possibility.

## Glycoprotein Hormone Family

Gonadotropins, in response to hypothalamic GnRH, are released from the pituitary and act on the gonads to regulate steroidogenesis and gametogenesis. Two GTHs, follicle-stimulating hormone (FSH) and luteinizing hormone (LH), together with TSH form a family of pituitary hormones (Figure 1). They are heterodimeric glycoproteins consisting of two subunits, an α-subunit and a unique β-subunit. These glycoprotein hormones (GPH) are believed to have evolved from a common ancestral molecule through duplication of β-subunit genes and subsequent divergence (27, 28). Two GTHs have been identified in all taxonomic groups of gnathostomes, including actinopterygians (29, 30), sarcopterygians (31), and chondrichthyans (32), but not in agnathans.

A single β-subunit of GTH was identified from the sea lamprey pituitary gland after extensive and exhaustive research for over 20 years (27, 33). However, the α-subunit of lamprey GTH is not found even in the lamprey genome (34). This is very strange fact, since a huge amount of physiological and morphological evidence has suggested the presence of GTH in the lamprey (33, 35–38). The lack of α-subunit of lamprey GTH makes difficulty to study the HPG axis in relation to GTH functions in the lamprey. The second form of β-subunit of pituitary GPHs as a candidate for TSHβ is not found in the lamprey genome (34), and thus the lamprey does not have TSH.

Recently, a fourth heterodimeric GPH has been discovered in the human genome and termed “thyrostimulin” due to its thyroid-stimulating activity (39). The thyrostimulin α-subunit, called glycoprotein α-subunit 2 (GPA2), is homologous but not identical to the common α-subunit (GPHα or GPA1). With the discovery of GPA2 and glycoprotein β-subunit 5 (GPB5, thyrostimulin beta) homologs not only in other vertebrates but invertebrates including fly, nematode, and sea urchin (40, 41), it is proposed that ancestral glycoprotein existed before the divergence of vertebrates/invertebrates, and that later gene duplication events in vertebrates produced the thyrostimulin (GPA2 and GPB5) and GTH/TSH [GPHα and GPHβ (LHβ/FSHβ/TSHβ)] (40) (Figure 1). The basal lineage of chordates such as tunicates and amphioxus contains GPA2 and GPA5 in their genome but not GPHα or GPHβ (2, 42–45). Lamprey also has GPA2 and GPB5 genes in addition to the canonical GTHβ (1, 33, 34, 42). At present, no information is available as to the presence of GPA2/GPB5 in the hagfish.

## Identification of Hagfish GTH

A single GPH, which comprises α- and β-subunits, was recently identified in the pituitary of the brown hagfish, *P. atami*, one of the Pacific hagfish (11) (Figure 2). Both subunits of GPH are produced in the same cells of the adenohypophysis, providing definitive evidence for the presence of a heterodimeric GPH in the hagfish. GPH increases at both the gene and protein levels corresponding to the reproductive stages of the hagfish (Figures 3C,D). Moreover, purified native GPH induces sex steroid release (estradiol-17β and testosterone) from cultured testis in a dose-dependent manner. From the phylogenetic analysis, the hagfish GPHα forms a clade with the gnathostome GPHαs. The hagfish GPHβ forms a clade with the TSHβs, however the bootstrap values are low and hagfishes evolved prior to the gnathostomes. The sea lamprey GTHβ also groups with the GPHβs but appears to be one of the outgroups of the LHβs. These results clearly show that the GPH identified in the hagfish acts as a functional gonadotropin, and hereafter it is referred as to GTH. Hagfish GTH is the earliest evolved pituitary GPH that has been identified in a basal vertebrate leading to the gnathostome GTH and TSH lineages.

## Feedback Regulation of Hagfish GTH Synthesis and Secretion

Gonadal steroid hormones and hypothalamic hormones play major roles in controlling the synthesis and release of LH and FSH in gnathostomes. Both positive and negative feedback effects of gonadal steroids have been demonstrated in teleosts, depending on modes of administration and reproductive stages of animals. In general, in sexually mature fish, sex steroids are considered to regulate gonadal maturation and recrudesce, whereas in juvenile fish, sex steroids are considered to regulate puberty. Thus, negative feedback effects of estradiol and testosterone are evident during the latter stages of gonadal development; specifically, it has been shown that gonadal removal increases LH secretion in salmon (46), goldfish (47), and African catfish (48). The observed increases in LH levels can be suppressed by treatment with estradiol, testosterone, or both. FSH is also controlled by steroid-dependent negative feedback loops in rainbow trout (49), salmon (50), and goldfish (51). The negative feedback effects of steroids may be mediated primarily at the levels of the hypothalamic GnRH neurons (52–54), because both *in vivo* and *in vitro* studies have shown that the expression of LHβ mRNA or FSHβ mRNA is often unchanged or increases following exposure to estradiol, testosterone, or both (49, 53, 55). However, in sexually immature teleosts, sex steroids appear to exert primarily a positive feedback effect that acts directly at the level of the pituitary and via effects on the GnRH system (55, 56). LH content and LH mRNA levels of the pituitary in juvenile fish increase in response to estrogens and aromatizable androgens (49, 57).

Estradiol treatment in the juvenile brown hagfish resulted in the marked accumulation of both immunoreactive (ir)-GTHα and ir-GTHβ in the pituitary (58). However, mRNA levels of GTHα and GTHβ in the pituitary were not, or only transiently, increased by the estradiol treatment (58). The latter results suggest that syntheses of both α- and β-subunits of GTH were not, or only transiently, affected by the estradiol treatment. Accordingly, the marked accumulation of both ir-GTH subunits could be attributed to the suppression of GTH secretion from the pituitary. From that study, the feedback effects of estradiol appeared to be inhibitory rather than stimulatory, and mediated by the possible suppression of the secretion of GTH from the pituitary in these juvenile hagfish. These conditions in juvenile hagfish resembled to those in adults, but not in juveniles, of teleosts (49, 53, 55). Such suppression of GTH secretion in the hagfish is probably regulated by the hypothalamic factors including GnRH, as mentioned below.

On the other hand, testosterone treatment in the juvenile brown hagfish had no effect on the staining intensities of the ir-GTHα and ir-GTHβ in the pituitary (58). Nevertheless, testosterone treatment resulted in the suppression of mRNA expressions of both GTHα and GTHβ in the pituitary (58). Therefore, testosterone probably acts to suppress both the synthesis and the secretion of GTH. This conclusion follows from the reasoning that if the secretion of GTH was not suppressed, the intensities of immunoreactions of both GTHα and GTHβ would have decreased due to decreased levels of mRNA expressions of both GTH subunits. Thus, it seems likely that estradiol and testosterone differ with regard to their roles in the regulation of synthesis and secretion of GTH in the pituitary of the hagfish.

## Plasma Levels of Sex Steroid Hormones in the Hagfish

Only a few studies exist regarding sex steroid hormonal profiles in relation to gonadal function in hagfish. Matty et al. (17) reported that estradiol and testosterone were measurable in the plasma of *Eptatretus stouti* using RIA; however, the observed levels of these steroids were near the lower limit of RIA sensitivity. Schützinger et al. (59) found using a more sensitive RIA that plasma estradiol content increased in relation to the stages of ovarian development in female Atlantic hagfish, *Myxine glutinosa*. Powell et al. (60, 61) also reported using *in vitro* organ cultured ovaries that the number of females with large eggs increased following estradiol peaks in January in *M. glutinosa*. Thus, estrogen seems to be involved in the ovarian development.

Plasma concentrations of estradiol, testosterone, and progesterone were examined with respect to gonadal development, sexual differences, and possible function of atretic follicles in the brown hagfish, *P. atami*, using a time-resolved fluoroimmunoassay (62). In females, plasma estradiol levels showed a significant positive correlation with ovarian development, while plasma testosterone and progesterone levels were highest in non-vitellogenic adults (62). Thus, our data on plasma estradiol levels in female *P. atami* were consistent with the results of Schützinger et al. (59) and Powell et al. (60). In another study, Yu et al. (63) demonstrated that the synthesis of hepatic vitellogenin was inducible by estrogens, estradiol, and estrone, in *E. stouti*. Based on these results, estrogenic control of ovarian development and hepatic vitellogenesis seemed to have arisen early in vertebrate evolution.

In males, no clear relationships were observed between plasma estradiol or testosterone concentrations and testicular development, while plasma progesterone concentrations showed a significant inverse relationship with testicular development (62). However, in that study data on sexually mature males with high incidences of spermatids or spermatozoa were lacking, since they were very few in our populations (62). Therefore, it is still possible to consider that estradiol and testosterone are involved in the regulation of male reproduction in hagfish. In support of this possibility, it is reported that purified native hagfish GTH induced secretion of estradiol and testosterone from cultured hagfish testes (11). Moreover, intraperitoneal administration of these steroids in juvenile hagfish affected on the GTH functions as mentioned above.

On the other hand, in relation to our failure to correlate plasma concentrations of estradiol or testosterone to testicular development, recent studies in the lamprey have emphasized the importance of non-classical steroids, such as androstenedione and 15α-hydroxylated sex steroids (15α-hydroxytestosterone and 15α-hydroxyprogesterone) in serving as functional androgens (64–67). Indeed, evidence demonstrating testosterone functionality in lampreys was scarce [see Ref. (68)], while androstenedione was found in substantial amounts within the testicular tissue of lampreys, and plasma and tissue levels of the hormone increased significantly in prespermiating male sea lampreys after injection of GnRH (66). In addition, prespermiating males implanted with androstenedione reached maturation significantly faster and exhibited larger secondary sex characteristics than placebo or non-implanted males (66). A receptor for androstenedione was recently described by Bryan et al. (66). 15α-Hydroxylated steroids are also suggested to be involved in the regulation of lamprey reproduction (67). Since hagfish gonads also produce substantial amounts of unusual androgens, such as 6β-hydroxy testosterone and 5α-androstane-3β, 7α, 17β-triol, as well as androstenedione (69–71), some of these steroids may act as functional androgens in the hagfish.

## Hypothalamic Factors Regulating the Gonadotropic Function of Hagfish

The synthesis and secretion of GnRH is the key neuroendocrine function in the hypothalamic regulation of the HPG axis. To date, two to three isoforms have been identified in representative species of all classes of gnathostomes and lampreys (1). GnRHs are also identified in tunicates (72), and several invertebrates belonging to lophotrochozoans [mollusk and annelid; (73, 74)], but not in the ecdysozoan lineages. On the other hand, adipokinetic hormone (AKH) has been identified as the ligand of the GnRH receptor of the insects, *Drosophila* and *Bombyx* (75). An AKH-GnRH-like neuropeptide has been identified in the nematode *C. elegans* (76). A comparative and phylogenetic approach shows that the ecdysozoan AKHs, lophotrochozoan GnRHs, and chordate GnRHs are structurally related and suggested that they all originate from a common ancestor (77).

In the hagfish, GnRH has not yet been identified, but previous chromatographic and immunohistochemical studies have suggested the presence of a GnRH-like molecule in the hypothalamic-neurohypophysial area (19, 20). Kavanaugh et al. (22) reported the seasonal changes in hypothalamic ir-GnRH contents in relation gonadal reproductive stages in the Atlantic hagfish (*M. glutinosa*). In *M. glutinosa*, a dense accumulation of GnRH-like immunoreaction was observed in the dorsal wall of the neurohypophysis with the use of antisera against chicken GnRH-II, salmon GnRH, lamprey GnRH-I, and lamprey GnRH-III (19, 21) (Figure 4). Neuronal cells containing ir-GnRH were found in the preoptic nucleus and the dorsal hypothalamic nucleus (20, 21). In another study, Osugi et al. (78) identified several PQRFamide peptides in the brain of the brown hagfish (*P. atami*). Based on *in situ* hybridization and immunohistochemistry, hagfish PQRFamide peptide precursor mRNA and its translated peptides were localized in the infundibular nucleus of the hypothalamus. Dense ir fibers were found in the infundibular nucleus and some of them were terminated on blood vessels within the infundibular nucleus. They further showed that one of the hagfish PQRFamide peptides significantly stimulated the expression of GTHβ mRNA in the cultured hagfish pituitary. The latter result clearly indicates that GTH functions of the hagfish pituitary are controlled by the hypothalamic factors.

Puzzling aspect of the hagfish hypothalamic-pituitary system is that there is no or little anatomical relationship between them. It is generally considered that the hypothalamic factors, such as GnRH, reach the adenohypophysis simply by diffusion (79, 80). However, the dorsal wall of the hagfish neurohypophysis, where ir-GnRH nerve fibers are terminated (Figure 4), is far from the adenohypophysis by the presence of the neurohypophysis itself. On the other hand, the blood vessels are richly distributed on the surface of the dorsal wall, and make the posterior hypophysial vascular plexus (14, 15). Although most blood in the posterior hypophysial vascular plexus enter the posterior hypophysial vein of the anterior cardinal system, several small vessels proceed from the dorsal wall to the adenohypophysis in *Eptatretus burgeri* (15). These small vessels may contribute the regulation of the adenohypophysial functions. A pair of small blood vessels from the hypothalamus also enters the posterior hypophysial vascular plexus (14). Together with the fact that some PQRFamide neuronal fibers terminated on the blood vessels within the hypothalamus (78), further studies are needed to understand the hypothalamic-pituitary system of the hagfish.

## Conclusion

Not only the pituitary gland but also all major adenohypophysial hormones and their receptors are considered to be vertebrate novelties. Since hagfish represent the most basal and primitive vertebrate that diverged over 550 millions years ago (81), they are of particular importance in understanding the evolution of the HPG axis related to vertebrate reproduction. Our data clearly show that the hagfish has a functional HPG axis similar to that of more advanced gnathostomes. It is strongly expected that the functional GTH found in hagfish pituitary helps to delineate the evolution of the complex HPG axis of reproduction in vertebrates. Furthermore, this HPG system likely evolved from an ancestral, pre-vertebrate exclusively neuroendocrine mechanism by gradual emergence of components of a new control level, the pituitary gland.

## Conflict of Interest Statement

The author declares that the research was conducted in the absence of any commercial or financial relationships that could be construed as a potential conflict of interest.
